# The Development of a Multidimensional Diagnostic Assessment With Learning Tools to Improve 3-D Mental Rotation Skills

**DOI:** 10.3389/fpsyg.2020.00305

**Published:** 2020-02-26

**Authors:** Shiyu Wang, Yiling Hu, Qi Wang, Bian Wu, Yawei Shen, Martha Carr

**Affiliations:** ^1^Quantitative Methodology Program, Department of Educational Psychology, University of Georgia, Athens, GA, United States; ^2^Department of Educational Information Technology, East China Normal University, Shanghai, China; ^3^Measurement and Statistics Program, Department of Educational Psychology and Learning System, Tallahassee, FL, United States

**Keywords:** mental rotation skills, learning program, diagnostic assessment, rotation strategy, longitudinal diagnostic model

## Abstract

This study reported on development and evaluation of a learning program that integrated a multidimensional diagnostic assessment with two different learning interventions with the aim to diagnose and improve three-dimensional mental rotation skills. The multidimensional assessment was built upon the Diagnostic Classification Model (DCM) framework that can report the binary mastery on each specific rotation skill. The two learning interventions were designed to train students to use a holistic rotation strategy and a combined analytic and holistic strategy, respectively. The program was evaluated through an experiment paired with multiple exploratory and confirmatory statistical analysis. Particularly, the recently proposed joint models for response times and response accuracy within dynamic DCM framework is applied to assess the effectiveness of the learning interventions. Compared with the traditional assessment on spatial skills, where the tests are timed and number correct is reported as a measure for test-takers' performances, the developed dynamic diagnostic assessment can provide an informative estimate of the learning trajectory for each participant in terms of the strengths and weaknesses in four fine-grained spatial rotation skills over time. Compared with an earlier study that provided initial evidence of the effectiveness of building a multidimensional diagnostic assessment with training tools, the present study improved the assessment and learning intervention design. Using both response times and response accuracy, thus current study additionally evaluated the newly developed program by investigating the effectiveness of two interventions across gender, country and rotation strategy.

## 1. Introduction

Spatial ability has long been considered as an important dimension of human intelligence through the studies in various populations and settings (e.g., Carroll, 1993; Eliot, 2012). It is an emerging area of interest to educators as spatial ability has been linked to better performance in mathematics and science achievement (Brownlow and Miderski, 2001; Thompson et al., 2013). The notion of spatial ability varies across studies. Different types of spatial skills have been measured including spatial perception, visualization and mental rotation (e.g., Perry, 2013; Weckbacher and Okamoto, 2014). Among these various spatial factors, mental rotation ability involves a cognitive visualization process to mentally rotate two-dimensional (2-D) or three-dimensional (3-D) objects. These two forms of mental rotation, particularly 3-D mental rotation, have been commonly associated with mathematics and science achievement (Voyer et al., 1995). Virtually, all 2-D and 3-D mental rotation tests involve presenting a target item and several solutions and the test taker has to mentally rotate the target to select the correct solution. One problem in this area is that little is known about the psychometric qualities of spatial skills tests or how or why students' performance differs as a function of test items. There are several possible causes of these problems. It may be the degree of rotation or manipulation needed, the complexity of the items, or the strategies used to solve items. Two strategies have been identified in literature: analytic/verbal and holistic (Glück et al., 2002). Holistic strategies involve rotating the entire object whereas analytic strategies involve matching parts of rotated objects to determine the correct answer. Both these two strategies can produce good outcomes but the holistic strategies are typically considered better examples of spatial processing and they seem to be more efficient and effective for more cognitive demanding spatial items; specifically items that require multiple, simultaneous rotations or that are complex (Wang and Carr, 2014). Some research studies also found that the combined analytic and holistic strategy might be more efficient than the sole holistic or analytic strategy, and it can decrease the gender difference (Stieff et al., 2014). Existing literature about mental rotation strategy also concluded that male and female students, Chinese Speakers and English Speakers may use different rotation strategies when solving spatial rotation questions (Weiss et al., 2003; Geiser et al., 2008; Li and O'Boyle, 2013; Li et al., 2014; Stieff et al., 2014).

While most studies in the literature focused on measuring the spatial ability or on investigating how the spatial ability is related to test-takers' characteristics, there has been a lack of research on investigating the factors that are related to the improvement of spatial skills. There are emerging evidence indicating that spatial ability can be improved (Uttal et al., 2013) and evidence that improving spatial skills results in improved mathematics (e.g., Cheng and Mix, 2014). Efforts to improve spatial skills have involved having participants practice on existing spatial skills tests or have involved extensive training in several aspects of spatial skills (e.g., isometric drawing). However, these instruction are time consuming and are not responsive to individual students' strengths and weaknesses.

This present study reported the development of a learning program that aims to improve mental rotation skills from a new perspective. This computer-based learning program integrates multiple multidimensional assessments with different learning interventions. Particularly, the embedded multidimensional assessments were built upon the Diagnostic Classification Model (DCM) framework. This is a family of restricted latent class models that can provide information concerning whether or not students have mastered each of a group of specific skills. These psychometric models have been used to design assessments that measure fine-grained skills or latent attributes across various domains, such as math skills (Bradshaw et al., 2014) and depression (Wang et al., 2019a). In addition to these applications of cross-sectional cognitive diagnostic assessment, the recently development of dynamic DCMs (e.g., Kaya and Leite, 2016; Li et al., 2016; Wang et al., 2017, 2018; Chen et al., 2018b; Zhan et al., 2019) enable the possibility of developing longitudinal cognitive diagnostic assessments to track skill learning and skill acquisition over time. This current study serve as the first attempt to develop the learning program within the longitudinal cognitive diagnostic assessment framework. Another important objective of this study is to evaluate the effectiveness of the developed learning program. Multiple exploratory and confirmatory analysis were conducted to evaluate the cognitive diagnostic assessment and learning interventions. Particularly, students' demographic information, such as gender, country and the rotation strategy, were collected and integrated with one of the recently developed dynamic DCMs, the joint model of response times and response accuracy (Wang et al., 2018, 2019b), to evaluate the learning interventions.

The rest of the paper is organized as follows. We first provide background on the test questions for measuring mental rotation skills, the Purdue Spatial Visualization Test: Visualization of Rotations (PSVT: R) and the revised PSVT:R. Second, we introduce the joint model of response times and response accuracy within dynamic DCM framework. This is followed by the description of the development of a new spatial rotation learning program. An experiment study is then presented to evaluate the learning program and understand students' learning behavior. We report the results from this experiment in the following section. Finally, the discussion section addresses implications for psychometrics and training mental rotation skills, limitations of the current study and future research study.

## 2. PSVT: R and Revised PSVT:R

The Purdue Spatial Visualization Test: Visualization of Rotations (PSVT: R), developed by Guay (1976), is one of the most popular tests that targets on measuring spatial visualization ability in 3-D mental rotation of individuals aged 13 years or older. This test has been frequently used in STEM education (Maeda and Yoon, 2013), and has shown in general good internal consistency reliability through several studies (Guay, 1976; Branoff, 2000; Alkhateeb, 2004). The PSVT: R consists of 30 items including 13 symmetrical and 17 non-symmetrical 3-D objects that are drawn in 2-D isometric format. Each item featured a reference object that had undergone a rotation. Test-takers then considered a new object and attempted to determine which of five options corresponded to the same rotation as the reference object. This test was revised by Yoon (2011) to correct the 10 figural errors identified by Yue (2006) and the format of the instrument was modified to avoid possible measurement errors. The revised test is named as revised PSVT:R. Since then, the revised PSVT:R has been used in several studies to investigate the psychometric properties of the test questions through Item Response Theory (IRT) Models (Maeda et al., 2013). They were also used to explore the association of the spatial ability of undergraduate students with gender, STEM majors and gifted program membership (Yoon and Mann, 2017).

## 3. DCMs and Dynamic DCMs for Response Times and Response Accuracy

Diagnostic Classification Model (DCM), or Cognitive Diagnosis Models (CDM), has emerged as an important statistical tool to help with diagnosing students' learning outcomes, such as skills and abilities that students have at the completion of a course or a learning program. These models assume that there are a number of pre-specified attributes measured by the assessment. A student's latent attribute profile is denoted by a multidimensional binary random vector with element 1 to indicate one possess a specific attribute and 0 to denote the lack of that particular attribute. In this way, DCMs can provide feedback regarding the measured skills. This allows for changes to be made in instruction, which can hopefully enhance students' learning. Research continues to document the benefits of DCMs as a framework for classifying students into educationally relevant skill profiles, and they have been used to study English-language proficiency (Templin and Hoffman, 2013; Chiu and Köhn, 2015), fraction subtraction (de la Torre and Douglas, 2004), pathological gambling (Templin and Henson, 2006), skills found in large-scale testing programs (Bradshaw et al., 2014; Li et al., 2015; Ravand, 2016), and Mental Rotation Skills (Culpepper, 2015).

The traditional DCMs are useful to classify attribute profiles at a given point in time. Recently research has begun to consider the role of DCMs to track learning and skill acquisition in a longitudinal fashion (Kaya and Leite, 2016; Li et al., 2016; Wang et al., 2017, 2018; Chen et al., 2018b; Zhan et al., 2019). In this type of research, the multidimensional binary latent skills for each student are assumed to be time-dependent and the purpose is to track the change of these binary skills overtime. Furthermore, in addition to the traditional product data, that is the response accuracy, the process data, such as the response times, are utilized to assess students' skill change over time. The joint model of response times and response accuracy (Wang et al., 2019b) used in this study is such an example. This joint model consists of a dynamic response model and a dynamic response time model. The dynamic response model includes a DCM as the measurement model to describe how test-takers respond to the assessment items with their attribute profiles at a given point of time, and a higher-order hidden Markov model that describes how the latent attribute profile changes from one time point to another, depending on the individual covariates (Wang et al., 2017). Like the traditional DCM, the dynamic DCM produces the output of the parameter estimation that quantify the psychometric properties for each item. It in addition can provide an estimate of students' learning trajectories in terms of the change of fine-grained skills over time. The estimated coefficients of the transition model from which can be used to identify the factors that are related to the transition probability and to evaluate the intervention. The dynamic response time model assumes students' latent speed on answering an item changes with the change of the latent attribute profile. It is thus directly connected with the dynamic response model through the latent attribute profile to provide additional information. The original work by Wang et al. (2019b) only considers the latent individual covariate in the dynamic response time model. In our study we will include students' demographic variables and problem-solving strategies to further investigate the between and within latent classes transitions. The details of this model are described in the Method section.

## 4. The Development of a New Spatial Rotation Learning Program

The new spatial rotation learning program reported in this study was developed on the basis of the findings from a previous research study (Wang et al., 2017). That old learning program was developed with the revised PSVT:R (Yoon, 2011) and consisted of five testing modules and four learning modules. Each of these modules contained 10 test questions. Four fine-grained mental rotation skills measuring the degree and direction of rotation were measured by test questions. That is (1) *x*90: 90° x-axis, (2) *y*90: 90° y-axis, (3)*x*180: 180° x-axis, and (4) *y*180: 180° y-axis. These four distinct yet related skills were identified to be measured by the revised PSVT:R through several previous studies (e.g., Maeda et al., 2013; Culpepper, 2015; Wang et al., 2017). To use this program, students first answered 10 questions in a testing module without any feedback to their answers then proceeded to a learning module in which they received feedback about their answers to the previous 10 questions and used a learning intervention to practice rotations. With such a design, test-takers need to finish 50 testing questions without feedback and to practice 40 additional questions with feedback and intervention. Positive findings of benefits of practice, an enhanced intervention, and the value of knowing some of the attributes, on the probability of making a transition to a master of a spatial skill, were demonstrated through a previous analysis (Wang et al., 2017). However, it was also found that a number of items had low psychometric qualities. This means that the students with low ability on spatial skills can easily guess the correct answer or the students with high spatial ability might easily miss the correct answer. These items provided less diagnostic information on measuring the spatial skills. Another finding was that students' performance in the 5th testing module is relatively lower than the 4th testing module, indicating there might be a fatigue factor due to the long testing and learning (it took about roughly 1 h and 15 min on average for students to finish this learning program). The following subsections provide details on the development of a new learning program based upon this old version of learning software.

### 4.1. The Learning Program Structure

**Figure 1:**
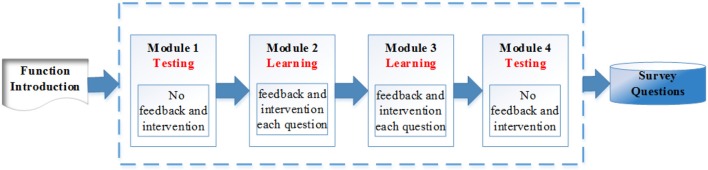
Spatial rotation learning program structure.

Compared with the old version, the whole structure of the learning program was redesigned to have two testing modules and two learning modules. The structure of the learning program is summarized by the flow chart in Figure 1. Specifically, this program starts with a testing module, followed by two consecutive learning modules, and finally ends with a testing module. The main purpose of module 1 and 4 is to accurately measure the four binary spatial skills at a given point in time. The two learning modules, model 2 and 3, aim to improve test-takers' mental rotation skills. The orders of these four modules are carefully designed thus are not exchangeable. The rationale of the design of these modules are summarized in section 4.2. Interventions are only provided in the learning modules. Each module contains 10 different questions, and they are selected based on various of item characteristics to reflect their functioning of assessing or improving the skills. A survey is provided at the end of the program to collect the test-takers' demographic information, the rotation strategy used by them during the test and their opinions about this learning program.

### 4.2. The Design of Module Blueprint

As described in the introduction, the learning program used the revised PSVT:R questions to measure four rotation skills. In fact, the original revised PSVT: R has 30 questions, and Wang et al. (2017) developed another 20 new items following the same item format so that a total of 50 questions are available to use in our study. Based on the learning program structure, 40 questions were selected from the existing 50 questions to assemble the four modules. These questions were selected based on different item characteristics, which can be measured from both a qualitative and quantitative point of view. The qualitative properties include the skill(s) measured by each item and the shape of the item. A very important component in the DCM based assessment, is a Q matrix (Tatsuoka, 1985), that specifies the rotation skill(s) measured by each item. The Q matrix is usually pre-determined by panels of subject-matter experts or estimated and validated based on the response data (e.g., Xu and Shang, 2017). In this study, we used the Q matrix in Wang et al. (2017), which was built based on the findings from Guay (1980) and Culpepper (2015). According to this Q matrix, each of the available 50 questions measures 1 or 2 skills. The shape of an item reveals the complexity in visualizing the 3-D object. The current 50 questions include symmetrical and non-symmetrical 3-D objects that are drawn in 2-D isometric format. The quantitative properties of the questions can be described by the difficulty and discrimination of the item. The difficulty of the PSVT: R items has been analyzed based on classical test theory and item response theory (Yoon, 2011; Maeda et al., 2013). The discrimination of the items describes how one item can discriminate/differentiate the students with low spatial ability from those with high spatial ability. Previous studies used the two parameter and three parameter logistic models (Maeda et al., 2013) and the deterministic input, noisy, “and” gate model (DINA; Junker and Sijtsma, 2001) to get item discrimination parameter estimation (Culpepper, 2015). In order to accurately measure students' mental rotation skills and to detect the possible learning effect, we design the two testing modules to have balanced and similar item quality. For the two learning modules, the main purpose is to help students improve their spatial skills and keep their motivation of using the learning intervention. Thus, the first learning module contains the relative easy items with simple shapes, with the purpose to minimize the side effect of lack of interest in learning due to frustration of providing too many wrong answers (as they are informed their answer is right or wrong in the learning module). The second learning module contains relatively harder and moderate to complex shape of items. In addition, the analysis on the learning data (Wang et al., 2017) revealed that the four attributes might have a hierarchical structure that implies that students who have mastery of 180 rotations should also be skilled at 90° rotation. In other words, the 90° rotation is the prerequisite for the 180° rotation. Thus, it's reasonable to guide students to learn the prerequisite skill first. Based on all above analysis, the finalized targeted properties of the items in the four modules are presented in Tables 1, 2. The next section summarizes the details of the selection of 40 questions based on both quantitative and qualitative analysis.

**Table 1:** The targeted properties of the items in four modules.

**Index**	**Module 1**	**Module 2**	**Module 3**	**Module 4**
Difficulty	Balanced	Easy	Moderate-high	Balanced
Discrimination	Balanced	Low-moderate	Moderate-high	Balanced
Shape	Balanced	Simple-moderate	Complex-moderate	Balanced

**Table 2:** The skill(s) measured by the number of questions across four modules.

**Attribute**	**Module 1**	**Module 2**	**Module 3**	**Module 4**	**Total**
*x*90	1	2	1	1	5
*x*180	1	2	1	1	5
*y*90	1	2	1	1	5
*y*180	1	3	2	1	7
*x*90, *y*90	2	1	2	2	7
*x*90, *y*180	2	0	1	2	5
*x*180, *y*90	2	0	2	2	6

### 4.3. Item Pre-analysis and Validation

#### 4.3.1. Quantitative and Qualitative Analysis

We conducted both qualitative and quantitative analysis to the 50 available questions from Wang et al. (2017) in order to select 40 from them to assemble the four modules based on the blueprint. For the quantitative aspect, using the data from a previous research study (Culpepper, 2015), a Rasch model was fitted to the 50 questions to produce the item difficulty parameters. Six raters with high spatial abilities were invited to rate the difficulty of each item, and their scores were highly positively correlated with the estimated difficulty parameters from the Rasch model, ranging from 0.89 to 0.94. The item discrimination, 1−*s*_*j*_−*g*_*j*_, is defined based on the Deterministic Input, Noisy “And” gate (DINA; Junker and Sijtsma, 2001) model, which describes how well an item can discriminate subjects who master all the required attributes for the item from subjects who do not master any of the required attributes. The larger the discrimination index, the more diagnostic information the item can provide. For the qualitative aspect, a spatial skill domain expert examined the shape of object in each question, and rated the complexity of the shape to the scale of 1–5. The higher the score, the more difficult for this object to be visualized as a 3-D object. Based on the characteristic of the 50 available questions, a heuristic automatic test assembly algorithm was developed to select 40 questions to assemble the four modules. This test assembly algorithm was developed by authors based on Armstrong's et al. (1992) Phase II algorithm to guarantee the four modules match the program blueprint (Tables 1, 2). The item positions in each module are in ascending order of item difficulty (from easy to hard).

#### 4.3.2. 3-D Model Building

**Figure 2:**
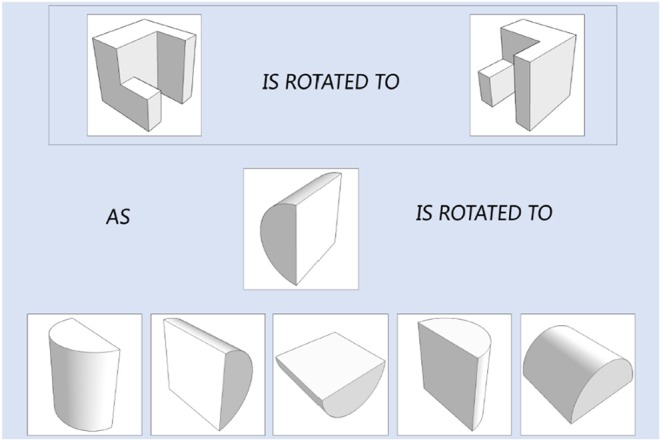
The 3D model for the objects/figures in an item.

The original PSVT: R presented the 3-D object in a 2-D isometric format. In the current study, in order to accurately measure the four fine-grained mental rotation skills that target on degree and direction of rotation only, all the objects in the 50 questions were reconstructed based on 3-D model building in computer and an example is presented in Figure 2. The 3-D models were constructed using 3ds Max 2016 developed by Autodesk. The questions in the testing modules and the learning modules are all like the one presented in Figure 2, which include a reference item that is rotated. Test-takers are presented a new object and they must select one answer from the five options that corresponds to the ending position of the new object, rotated the same way as the reference item. In the testing module, test-takers are not informed about whether their questions are correct or wrong. And in the learning module, they are informed immediately about the correct answer correct or not after each question. In addition, in the learning module, test-takers have the chance to interact with a learning intervention to practice rotation. The next subsection describes the intervention design.

### 4.4. Learning Intervention Design

#### 4.4.1. Two Learning Interventions

**Figure 3:**
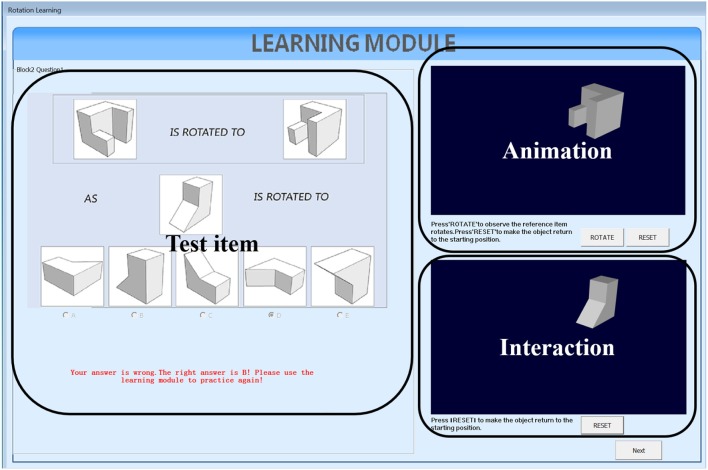
Non-colored intervention.

**Figure 4:**
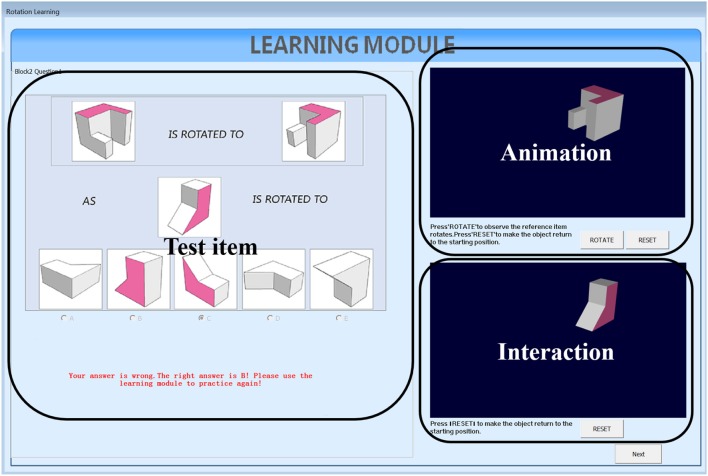
Colored intervention.

We developed two types of learning interventions by using C++ with Visual Studio 2012. One version is animation plus interaction as shown in Figure 3. The left panel of Figure 3 shows the testing items, the top panel on the right shows animation of rotating the reference object from the initial position to the final position and the bottom panel on the right allows users to rotate the testing object from the initial position to the final correct position by following the rotation path from the reference one. This type of intervention intends to train test-takers with the *holistic strategy*. The other intervention has the same functions as the first one and with an additional coloring feature (Figure 4). One of the facets of both reference and testing objects in three panels was draw with pink color. This is designed to help test-takers figure out the final position of the testing object by mapping the pink facet in the initial position to its final position. This coloring is more like training the test-takers using an analytic strategy. Combined with the rotation functions from the right panels, the second intervention intends to train test-takers with a *combined analytic* and *holistic strategy*.

#### 4.4.2. Learning Routine

In both versions of intervention, test-takers follow the same learning routine of three steps: (a) solve the displayed testing question on the left panel with the top and the bottom right panels invisible, and hit the check answer button to receive the feedback; (b) the two panels on the right are then displayed and test-takers can press the rotate button on the top right panel to watch the rotation animation of the reference object; (c) test-takers need to further rotate the testing object in the bottom right panel to the correct position. During this process, test-takers are allowed to repeat step (b) and (c).

## 5. Method

### 5.1. Experiment Study

#### 5.1.1. Sample

The participants in this experiment were undergraduate students, 18 years or older, enrolled in three Universities, one in United States and two in China. Recruiting participants in two countries can help us investigate whether there is cultural difference in terms of learning spatial skills. In order to get enough sample size, participants in both countries were recruited by two ways. The first was to recruit participants through the Educational Psychology or Psychology Research Participant Pool. Participants from this source were rewarded 1 course credit after completing this study. The second was to recruit participants through flyers and email announcement. For those participants, they were paid with a base amount of money and can earn additional amount of payment for each question answered correctly. From Spring 2017 to Summer 2017, recruitment through the above two approaches yielded 585 participants. Because of the various sources of recruiting participants, we fitted a mixture learning model (Zhang and Wang, 2018) to exclude some participants who were identified to be not engaged in the experiment. These participants' response data did not reflect the measured latent attributes and cannot be used to evaluate the learning program. Through this procedure, a total of 548 students were included for final data analysis.

#### 5.1.2. Procedures and Variables

The experiment was conducted in the computer lab in each University. The two types of learning interventions (colored and non-colored) that corresponding to the combined and holistic rotation training strategy were randomly assigned among the participants. Before starting the learning program, the participants first watched the instruction about how to use the learning program. Researchers in the computer lab also gave directions on how to use the program and they were available to answer questions during the experiment. Participants were informed that they had as much time as they wanted to complete this assessment. They were told that this study was conducted to understand how people solve and learn spatial rotation tasks. The participants who received the payment instead of the course credit were informed that the payment were based on the number of questions answered correctly. On average, it took 30 min for the participants to finish the experiment.

The participants' binary responses and their response time to each of the 40 test questions were recorded by the software directly. In addition to these response data, a survey after each participant completed the experiment collected participants' demographic information, such as gender (female and male), the country (China and US), and the strategy participants used to solve the questions (Analytic, Holistic and Hybrid). The information about the rotation strategy used by each participant was collected based on a self-report question in the survey. These covariates will help us further evaluate the developed diagnostic assessment and learning interventions across different populations. In the survey, participants also provided their opinions about whether the learning module can help them learn rotation skills on the Likert scale [1 (not helpful)–5 (very helpful)].

### 5.2. Exploratory Statistical Analysis

#### 5.2.1. Descriptive Statistic

Descriptive statistics, such as the number of participants (*N*), the means and standard deviations of the module scores in the learning program and the module completion time, are presented in Tables 3, 4 by participants' characteristics, such as gender, country, strategy used to solve the problem and the intervention. A slightly increase of the mean score in module 4 compared with module 1 can be observed. The module 3 contains the items that are most difficult while module 2 consists of the items that are easiest among the four modules. Thus, the average score in module 3 is the lowest and the average score in module 2 is the highest among the four modules across different groups. Note that, we can hardly eyeball the “growth” based on the descriptive statistics in different modules, as each module has different item difficulty. In addition, the evaluation of the learning program should target on the population who have relatively low spatial rotation skills. However, in order to recruit participants as many as possible in a short time, we did not conduct a separate pretest to exclude the participants who already had a high spatial rotation ability. Thus, the final 548 sample may mix a proportion of participants who do not need to improve their spatial skills. Fortunately, the joint learning models presented in the later section can consider the item difficulty and help us identify the participants who already mastered the four skills in the very beginning. In terms of the completion time, participants spent least time on completing module 2, which is consistent with that module 2 is the easiest one. Though module 3 contains the most difficult items, participants on average spent less time on it compared with the module 1, which is relatively easier. This might be due to the warm-up effect for module 1, in which participants were still not very familiar with the questions or due to the improvement of their spatial rotation skills so that they can apply those skills more quickly in module 3. The distribution of participants over country and intervention are roughly balanced, while for gender and rotation strategy, the distributions are unbalanced. The large proportion of the female participants and combined rotation strategy used by participants are mainly due to our convenience sampling procedure and self-report of the strategy in the survey.

**Table 3:** Descriptive statistics for 548 participants (response scores).

**Variable**			**Module score**			
		***N***	**1**	**2**	**3**	**4**
Gender	Female	401	7.00 (1.87)	8.76 (1.30)	6.34 (2.02)	7.15 (1.85)
	Male	147	7.76 (1.80)	9.16 (1.05)	6.71 (1.95)	7.70 (1.85)
Country	US	223	6.94 (1.85)	8.63 (1.37)	5.74 (1.92)	7.27 (1.82)
	China	325	7.38 (1.89)	9.03 (1.13)	6.91 (1.92)	7.31 (1.89)
Strategy	Analytic	62	7.37 (2.03)	8.84 (1.16)	6.56 (2.09)	7.52 (1.80)
	Holistic	63	6.71 (2.02)	8.79 (1.05)	6.11 (1.89)	7.14 (1.88)
	Combined	423	7.25 (1.83)	8.88 (1.29)	6.46 (2.01)	7.29 (1.87)
Intervention	Color	264	7.07 (2.00)	9.06 (1.14)	6.58 (1.87)	7.13 (1.91)
	Non-color	284	7.32 (2.00)	8.68 (1.32)	6.30 (2.11)	7.44 (1.81)

*The numbers in the brackets are the standard deviation. The total score for each module is 10*.

**Table 4:** Descriptive statistics for 548 participants (response time).

			**Module completion time (minute)**			
		***N***	**1**	**2**	**3**	**4**
Gender	Female	401	8.76 (4.30)	4.71 (2.12)	7.23 (3.49)	6.84 (3.30)
	Male	147	8.13 (3.84)	4.06 (1.94)	6.52 (3.21)	5.98 (2.53)
Country	US	223	7.17 (3.88)	3.84 (1.76)	6.15 (3.28)	6.15 (3.27)
	China	325	9.56 (4.12)	5.01 (2.17)	7.64 (3.40)	6.92 (2.99)
Strategy	Analytic	62	9.55 (4.80)	4.96 (2.45)	7.99 (4.33)	6.67 (3.24)
	Holistic	63	7.27 (3.10)	3.74 (1.28)	5.78 (2.62)	5.48 (2.45)
	Combined	423	8.64 (4.19)	4.59 (2.11)	7.08 (3.33)	6.77 (3.18)
Intervention	Color	264	8.37 (4.07)	3.99 (1.75)	6.21 (3.15)	6.68 (3.29)
	Non-color	284	8.80 (4.29)	5.05 (2.25)	7.81 (3.49)	6.55 (2.98)

*The numbers in the brackets are the standard deviation*.

#### 5.2.2. Clustering Analysis on Items

**Figure 5:**
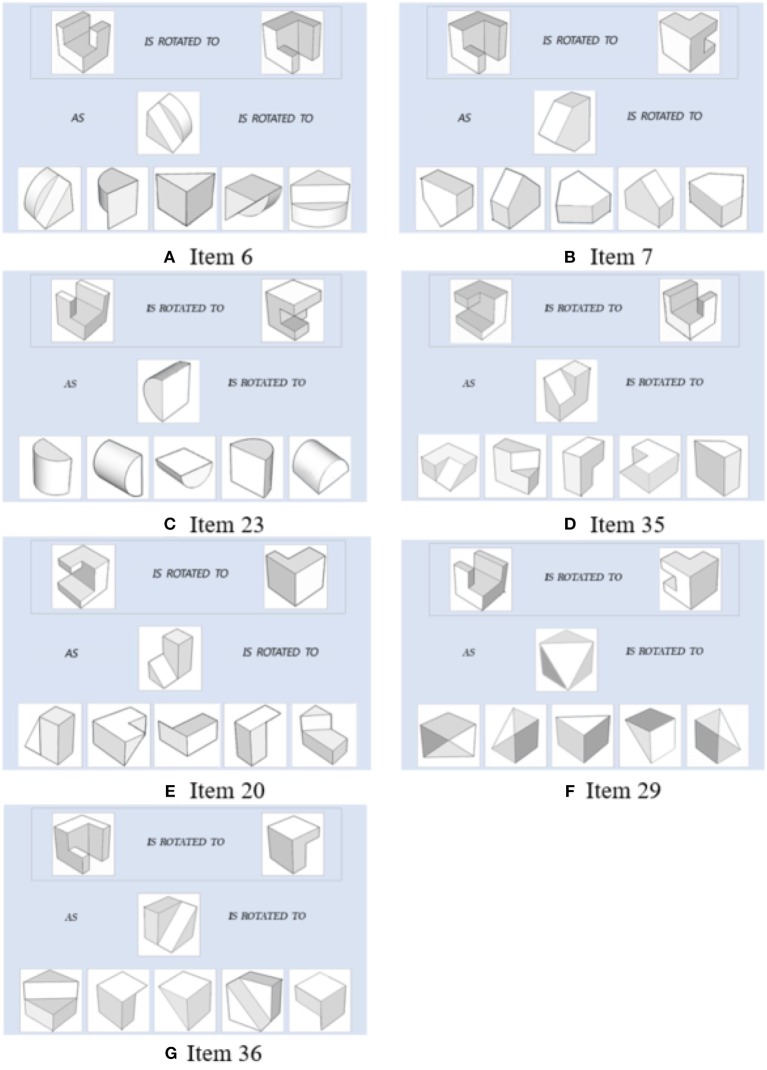
Items measuring *x*90 and *y*90. Items 6,7,23, and 35 are clustered with simple items. Items 20, 29, and 36 are clustered with other complex items.

A very important component used in the joint model of response times and response accuracy is the Q matrix, which gives the information on which attributes are measured by each item. The previous research studies on Q matrix estimation or validation are in general conducted in through a confirmatory way that assumes students' responses follow a specific DCM (e.g., Xu and Shang, 2017). In this study, we conduct exploratory clustering analysis on items, using not only responses but also response times. The item group results from the cluster analysis can be used to compare with the existing Q and further valid it in the future. One clustering algorithm that accounts for both continuous and categorical data is K-prototype (Huang, 1997). We apply this method to group items in each module based on the categorical responses and continuous response times in which. The number of clusters, *M*, is determined by the Silhouette index (Rousseeuw, 1987), which is commonly used in clustering analysis (e.g., Rendón et al., 2011; Hämäläinen et al., 2017). This index measures the similarity of an item to its cluster compared to other clusters and its value ranges from −1 to 1. A value of 1 is ideal as it suggests that data point is far away from other clusters. On the contrary, value of −1 is not preferred because it indicates that the data point is closer to other clusters than to its own. In our study, we use the Global Silhouette value, which is the average of the total silhouette values for all items of each cluster, to determine the number of clusters (Bolshakova and Azuaje, 2003). For all four modules, the average Silhouette values were highest when *M* = 2. Based on this, we group items into two clusters for each module. Note that the items can be in general classified as two types based on the Q matrix. One are simple items which measure only one attribute, the other are complex items which measure more than one attributes. The clustering results from K-prototype indicated that for each module, all simple items were grouped together and most complex items were grouped into another cluster. We note that four complex items, item 6 and 7 in module 1, item 23 in module 3, and item 35 in module 4, were grouped with simple items instead. Based on the current Q matrix, these four items all measure attributes *x*90 and *y*90. To explore the reason of mismatching of these four items, we compared them with item 20, 29, and 36, which also measure attributes *x*90 and *y*90. It was found that the 3D objects in item 6, 7, 23, and 35 are in relative simple shapes compared with those for item 20, 29, and 36, as shown in Figure 5. Moreover, the response accuracy and response times on item 6, 7, 23, and 35 were closer to simple items than the complex items measuring the same attributes. For example, the mean response time for item 35, simple and complex items in module 4 are 36.96, 27.04, and 50.33 s, respectively, and the mean correct response proportion for these three groups are 0.7, 0.76, and 0.54, respectively.

### 5.3. Confirmatory Statistical Analysis

#### 5.3.1. The Joint Model of Response Time and Response Accuracy

In a longitudinal set up, such as the one in our study, the multidimensional binary latent skills for an individual *i* at time *t* are denoted as $\alpha_{i}(t)=(\alpha_{i1}(t),\ldots ,\alpha_{iK}(t){)}^{\prime }$, with *t* indexes time and *k* = 1, …, *K* indexes attributes and α_*ik*_(*t*) = 0 indicating non-mastery and 1 meaning mastery. Test-takers' responses are also time dependent, and the *i*_*th*_ test-taker's responses to *J* questions at time *t* can be denoted as **Y**_*i*_(*t*) = (*Y*_*i*1_(*t*), …, *Y*_*iJ*_(*t*)), with *Y*_*ij*_(*t*) = 1 if the test-taker responded correctly to item *j* at time *t*, and 0 otherwise. In addition, the computer records the response time on completing each test question for each test taker, denoted by *L*_*i*_(*t*) = (*L*_*i*1_(*t*), …, *L*_*iJ*_(*t*)). Both **Y**_*i*_ and *L*_*i*_ are used to provide an estimate of each test-taker's learning trajectory in terms of the change of fine-grained skills over time based on responses and also an estimate of their initial latent speed and the change of the speed due to the latent attribute profile and other covariates.

Specifically, the joint model proposed by Wang et al. (2019b) consists of a dynamic response model and a dynamic response time model. The dynamic response model includes two components. For each time *t*, a measurement model is used to model *P*(*Y*_*ij*_(*t*)|α_*i*_(*t*)). An example is

(1) $$\begin{array}{l} P\left(Y_{ij}\left(t\right)=1|\alpha_{i}\left(t\right),s_{j}\left(t\right),g_{j}\left(t\right)\right)=\left\{ \begin{array}{cc} 1-s_{j}\left(t\right) & \text{if }\alpha_{i}\left(t\right)≽q_{j},\\ g_{j}\left(t\right) & \text{otherwise}, \end{array}\right. \end{array}$$

where q_*j*_ denotes the skills measured by item *j*. The notation ≽ indicates the test-taker *i* with latent attribute profile α_*i*_(*t*) has mastered all the required skills for item *j* at time *t*. The model describes by Equation (1) is the DINA model, which uses two parameters to describe the correct response probability to each item given the test-takers' latent profile and the required skills for that item. For example, if the test-taker's latent profile at this time is (1, 1, 0, 0)′, meaning he mastered the 90° rotations along the *x* and *y* axes. If item *j* only requires 90° rotation along *x* axis, then this test-taker has probability 1 − *s*_*j*_(*t*) to answer this item correctly. The term *s*_*j*_(*t*) is the slipping probability that refers to the probability that the test-taker misses item *j* at time *t* that his level of mastery suggests he would be expected to answer correctly to it. In the other case, if this item *j* requires the 180° rotation along *x* axis, and this test-taker does not master this required skill, then he has the probability *g*_*j*_(*t*) to answer this item correctly. This probability, *g*_*j*_(*t*), is called the guessing probability that describes the chance that the test-taker correctly answers a question that his level of mastery would suggest he should not. The DINA model is a very simple DCM model with a conjunctive structure. It assumes only two correct response probabilities for each item. Many popular CDMs, such as the models assumes a compensatory structure or in more general forms can also be a candidate for this measurement portion. In the subsequent section, we will conduct a measurement model selection procedure to determine the most appropriate measurement model for our data.

The second component in the dynamic response model is a transition model, which describes how the latent attribute profile changes from one time point to another. This transition model assumes non-decreasing skill trajectories and conditional independence of attribute-wise transitions given the previous attribute pattern, and hence, it focuses on modeling the transition of each skill from non-mastery (0) to mastery (1), depending on several latent and observed covariates. To model the transition probability, we first assume the transition of an unlearned skill from 0 to 1, depends on a general learning ability. This general ability is denoted as a latent continuous variable for each test-taker *i* as θ_*i*_ and the number of learned skills. In addition, as one of the primary objectives of this study is to compare the two types of interventions on improvement of the rotation skills across gender, country and problem solving strategy, the variables reflect this information are also included in this model. In summary, the covariates we considered in the transition model are the main effects of general learning ability θ, the mastered skill(s), the gender, country, intervention, rotation strategy, as well as the two-way interactions between intervention and gender, intervention and country and intervention and rotation strategy. It can be written as,

(2) $$\begin{array}{l} \;logit\left(P\left(\alpha_{ik}\left(t+1\right)=1|\alpha_{ik}\left(t\right)=0\right)\right)\\ =\lambda_{0}+\lambda_{\theta }\theta_{i}+\lambda_{\alpha }\underset{l\neq k}{\sum }\alpha_{il}\left(t\right)+\lambda_{g}*{\text{gender}}_{i}\\ +\lambda_{c}*{\text{country}}_{i}+\lambda_{I}*IV_{i}+\lambda_{st1}*{\text{Strategy}}_{1i}+\lambda_{st2}*{\text{Strategy}}_{2i}\\ +\lambda_{gI}*{\text{gender}}_{i}*IV_{i}+\lambda_{cI}*{\text{country}}_{i}*IV_{i}\\ +\lambda_{Ist1}*IV_{i}*{\text{Strategy}}_{1i}+\lambda_{Ist2}*IV_{i}*{\text{Strategy}}_{2i}. \end{array}$$

Here $\underset{l\neq k}{\sum }\alpha_{il\left(t\right)}$ quantifies the number of mastered skills at time *t*. *IV*_*i*_, gender_*i*_ and country_*i*_ are dummy variables representing the two levels of each categorical variable. The Strategy_1_ and Strategy_2_ are the two dummy variables denoting the three levels of the rotation strategies used by the test-takers. Each of the component in the coefficient vector $\lambda ={\left(\lambda_{\theta },\lambda_{\alpha },\lambda_{g},\lambda_{c},\lambda_{I},\lambda_{st1},\lambda_{st2},\lambda_{gI},\lambda_{cI},\lambda_{Ist1},\lambda_{Ist2}\right)}^{{}^{\prime }}$ describes how the corresponding covariate influences the odds of skill transition from 0 to 1. These estimated values can help us evaluate the designed learning program.

Finally, the dynamic response time model is built based on a log-normal distribution. That is, the model assume the log of response time on each question follows a normal distribution, where the mean depends on a time intensity parameter(γ_*j*_), the test taker's initial latent speed (τ_*i*_), and the covariates that may influence the speed during the learning process. The variance of the distribution is characterized by a time discrimination parameter (*a*_*j*_). The log-normal response time model is chosen based on the analysis from a previous research study that used the same experiment data set (Zhang and Wang, 2018). The key part of the dynamic response time model is on defining a latent covariate that connects the latent attribute profile and identifying several observed covariates that may impact the speed. In our case, we use a fixed effect model as the following specific form.

(3) $$\begin{array}{l} log\left(L_{i}j\left(t\right)\right)\sim N\left(\gamma_{j}-\left(\tau_{i}+\underset{h=1}{\sum }\phi_{h}Cov_{h}\right),\frac{1}{a_{j}}\right). \end{array}$$

The quantity $\underset{h=1}{\sum }\phi_{h}Cov_{h})$ in Equation (3) describes the different covariates that may impact the speed. Specifically,

(4) $$\begin{array}{l} \underset{h=1}{\sum }\phi_{h}Cov_{h}=\phi_{\alpha }G\left(\alpha_{i},{\text{q}}_{j}\right)+\phi_{g}*{\text{gender}}_{i}\\ \;+\phi_{c}*{\text{country}}_{i}+\phi_{I}*IV_{i}+\phi_{st1}*{\text{Strategy}}_{1i}\\ \;+\phi_{st2}*{\text{Strategy}}_{2i}\\ \;+\phi_{gI}*{\text{gender}}_{i}*IV_{i}+\phi_{cI}*{\text{country}}_{i}*IV_{i}\\ \;+\phi_{Ist1}*IV_{i}*{\text{Strategy}}_{1i}+\phi_{Ist2}*IV_{i}*{\text{Strategy}}_{2i}. \end{array}$$

The *G*(α_*i*_, q_*j*_) is the latent covariate that connects the learning trajectory α_*i*_ with the response time model. We define *G*(α_*i*_, q_*j*_) = 1 is α_*i*_(*t*) ≽ q_*j*_ and 0 otherwise. In this way, this covariate classify the change of speed into 2 classes on each item. The other observed covariates in (4) are the same as those in the transition model (2), and we are interested in investigating whether those covariates can give us additional information on the respond speed after controlling the latent learning trajectory. Such information are useful to evaluate the developed learning interventions.

In summary, the confirmatory joint model of response times and response accuracy can produce a learning trajectory for each test taker. In our case, if the latent profile is described based on the order of *x*90, *y*90, *x*180, and *y*180, and for a participant with the initial latent profile as (0, 1, 0, 0)′, indicating one masters only the 90° rotation along *y* axis, then joint model can provide an estimate of the latent profile after each stage of the learning program. The improvement of a specific rotation skill can be observed as the change from non-mastery (0) to mastery (1). In addition, the estimated coefficients in the transition model (λs) and dynamic response model (ϕs) can be used to evaluate the effectiveness of the learning program cross different populations defined by various latent and observed covariates.

##### 5.3.1.1. Selection of the response measurement model

Before fitting the joint model, we first need to select the appropriate measurement model for responses. The models we consider are DINA, the deterministic-input, nopisy-or-gate model (DINO; Templin and Henson, 2006), the reduced reparameterized unified model (RRUM; Hartz, 2002), linear logistic model (LLM; Maris, 1999), the additive CDM (ACDM; de la Torre, 2011), and generalized DINA (G-DINA; de la Torre, 2011). These models are the representatives of DCMs that either have conjunctive/compensatory assumptions or belong to a family of models that have more general assumptions. To select the most appropriate model, we performed both test-level and item-level model selection procedures, treating each module as a mini test. These procedures were conducted using package *GDINA* (Ma et al., 2019) with Expectation-Maximization algorithm in R version 3.5.1 (R Core Team, 2018). For the test-level model selection, the Akaike information criterion (AIC) and Bayesian information criterion (BIC) were used. Table 5 represents the values of AIC and BIC for multiple models at module level. For module 2–4, both AIC and BIC suggest that the best measurement model is DINA. However, for module 1, AIC suggests the GDINA and BIC suggests the DINO. The BIC value from the DINA model is very close to the DINO. For the item-level model selection, we apply the Wald test (de la Torre and Ma, 2016; Ma and de la Torre, 2016) to determine the most appropriate model for each item. The reduced models with *p*-values less than the pre-specified α level were rejected. If all reduced models were rejected for an item, the GDINA model was used as the best model; if more than one reduced models were retained, the reduced model with the largest *p*-values is selected as the most appropriate model with prioritizing DINA and DINO. Before doing that, we note that there are in fact 21 items that measure only one attribute. For these items, all types of DCMs are equivalent to the DINA model. The For the rest 19 items, the Wald test suggests that DINA model fits best for 12 of them. Other reduced models, such as RRUM, ACDM, and DINO, fit best for the rest 7 items. The details of the Wald test rest are summarized in Table 1A in Appendix. Both the test-level and item-level results suggest the DINA model fits most of the test questions, and also given its simple format, we choose to use the DINA model as the measurement model in the joint model.

**Table 5:** Model-data fit indices.

	**Module 1**		**Module 2**		**Module 3**		**Module 4**	
**Model**	**AIC**	**BIC**	**AIC**	**BIC**	**AIC**	**BIC**	**AIC**	**BIC**
DINA	5996.65	6147.37	3629.94	3780.66	6452.84	6603.56	5485.19	5635.91
DINO	5993.90	6144.62	3632.55	3783.27	6469.02	6619.74	5498.81	5649.53
GDINA	5979.02	6181.41	3633.79	3793.12	6464.39	6658.17	5498.36	5700.76
RRUM	5985.95	6162.51	3631.89	3786.91	6458.71	6630.96	5496.97	5673.53
LLM	5982.26	6158.81	3631.75	3786.78	6459.18	6631.43	5498.22	5674.77
ACDM	5982.03	6158.59	3631.84	3786.87	6458.79	6631.04	5499.01	5675.57

##### 5.3.1.2. Model convergence result

**Figure 6:**
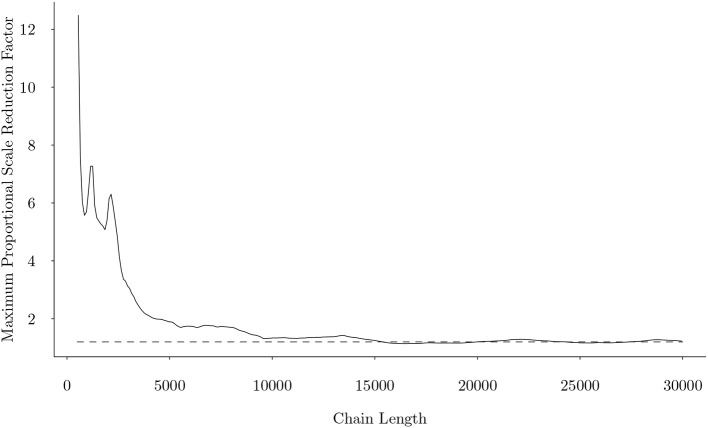
The maximum univariate Gelman-Rubin proportional scale reduction factor from the joint model as a function of number of iterations, when uniform initial attribute patterns were used. Dotted line represents the cutoff of 1.2.

The joint model was calibrated through a Metropolis-Hastings within Gibbs Sampler (Wang et al., 2019b) through R (R Core Team, 2018). The MCMC chain convergence was evaluated by the Gelman-Rubin proportional scale reduction factor (PSRF) (Gelman and Rubin, 1992), commonly known as $\hat{R}$. Based on this criterion, this fitted model converged quickly as that shown in Figure 6. We can observe that after about 15,000 iterations, the maximum Gelman-Rubin proportional scale reduction factor among all parameters fell below 1.2, indicating that parameter estimates have stabilized.

#### 5.3.2. Item Analysis for Testing and Learning Modules

##### 5.3.2.1. Item parameters

**Figure 7:**
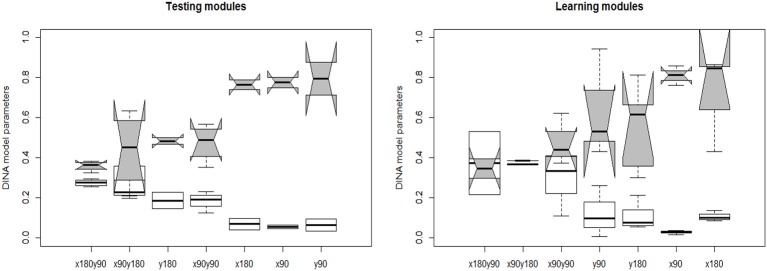
The estimated DINA model item parameters. The white whisker diagram represents the slipping parameters *s* and the gray ones represent the guessing parameters *g*.

**Figure 8:**
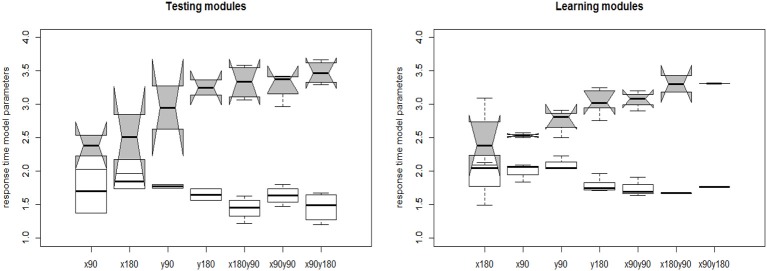
The estimated response time model item parameters. The white whisker diagram represents the time discrimination parameter *a* and the gray ones represent the time intensity parameters γ.

The joint model of response accuracy and response times is able to estimate two types of item parameters for each item: the slipping and guessing parameters from the DINA model and the item discrimination and item intensity parameters from the log-normal response time model. The distribution of the estimated guessing and slipping parameters for the 40 rotation questions are summarized in terms of boxplots in Figure 7. The items in the testing modules and learning modules are presented separately to better compare their characteristics. In each of the boxplot, the x axis denotes the item type in terms of the attributes measured by that item and the y axis denotes the estimated parameter value for an item with certain measured skills. The distribution of the slipping and guessing parameters had the similar pattern for the items in the testing modules (module 1 and 4) and learning modules (module 2 and 3). Specifically, the items require only one simple skill, such as *x*90 or *y*90, tend to have large guessing parameters and small slipping parameters. The items require one complex skill, such as *y*180, or two skills, have small guessing parameters and large slipping parameters. The variation of the same type of item parameters is larger for the items in the learning module than that in the testing modules. Similarly, the distribution of the estimated time intensity and time discrimination parameters of the 40 items are documented in Figure 8. Again, the distribution of these parameters had the similar pattern in the testing and learning modules. That is, the items require one simple skill tend to have small time intensity parameters and large time discrimination parameters. The items require one complex skill or two skills tend to have large time intensity parameters and small time discrimination parameters. The average values of DINA model parameters and response time model parameters for each module are presented in Table 6. The distribution of these parameters are relatively consistent with the test assembly requirement presented in Table 1. That is, the two testing modules (module 1 and 4) were assembled with items that had balanced item quality, while the two learning modules (module 2 and 3) were designed based on their corresponding learning functions.

**Table 6:** The mean item parameters for each module.

**Function**	**Modules**	**s**	**g**	**1−*s*−*g***	**a**	**γ**
Testing	Module 1	0.182	0.560	0.259	1.657	3.288
	Module 4	0.193	0.514	0.293	1.560	2.970
Learning	Module 2	0.065	0.757	0.178	1.978	2.680
	Module 3	0.272	0.418	0.310	1.757	3.093

**Figure 9:**
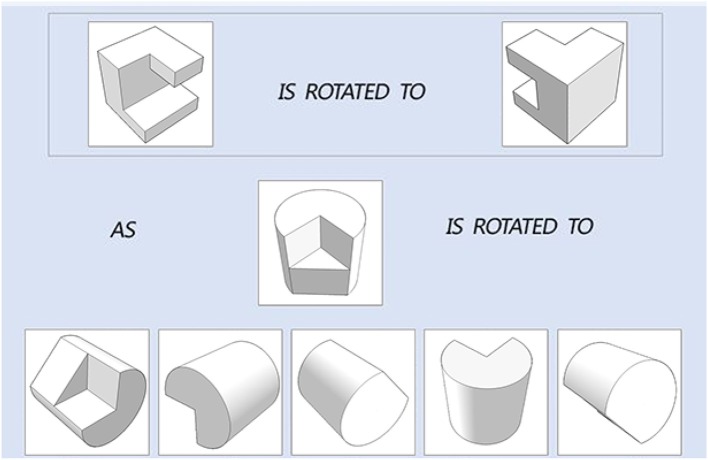
The item (ID: N10) with largest guessing parameter *g*.

**Figure 10:**
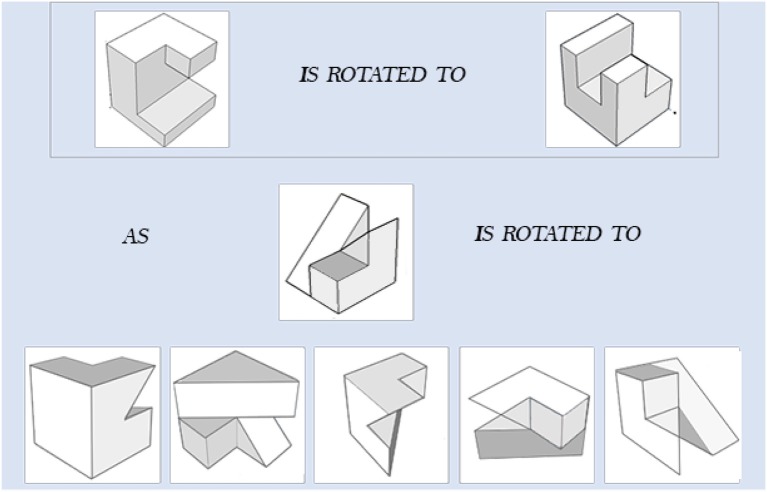
The item (ID:30) with the largest slipping parameter *s*.

Next, we focus on the analysis with some items that were identified to have extreme item parameters. The item with the largest guessing parameter, which is a new item created based on the revised PSVT: R, is presented in Figure 9. This item is also the one that has the largest item discrimination parameter. The reference object in this item measures the 180° rotation along *x* axis. If the participants can recognize the rotation is along the *x* axis, they can easily exclude the four distractors and select the correct option (the 4*th* one). It may be due to this reason, this item has the largest guessing probability. The distractors of this new item need to be further refined in the future to better diagnose the test-takers' rotation skills. For the current learning program, this item is the second question of the first learning module, thus the main function is to help test-takers learn the rotation. The item with the largest slipping parameter is presented in Figure 10. It has a relatively large time intensity parameter as well. It measures 180° rotation along the *x* axis, and 90° rotation along the *y* axis, and the object in this item has the most complex shape. Again, this item may not have a good diagnostic function. However, in the current learning program, it is the last question in the second learning module, and the main purpose is to improve test-taker's rotation skills.

##### 5.3.2.2. Reliability analysis for two testing modules

A reliability analysis was conducted to evaluate the two testing modules (module 1 and 4). In our study, classification consistency index (CCI; Cui et al., 2012) was chosen to estimate the test reliability. The CCI is the probability of classifying a randomly selected examinee consistently according to two administrations of a test. The range of CCI is between 0 and 1, and a higher values indicate a larger reliability. The CCI for module 1 and module 4 are 0.729 and 0.931.

### 5.4. Evaluation the Effectiveness of the Learning Program

The learning program is evaluated using the joint model results. The rest of this section reports the results from the dynamic response model and dynamic response time model portion of the joint model.

#### 5.4.1. Dynamic Response Model Result

The estimated coefficients from the transition model are documented in Table 7. Based on the 95% creditable interval, only the general learning ability θ and the learned skills were statistically related to odds of the transition probability. This indicates that after controlling the latent variables and based on the response accuracy across different time points, the two learning interventions (colored and non-colored) have the same effectiveness in improving the spatial rotation skills across gender, country, and the rotation strategy.

**Table 7:** The estimated coefficients from the transition model.

				**95% credible interval**	
**Variable**	**Notation**	**Mean**	***SD***	**Lower bound**	**Upper bound**
θ	λ_θ_	2.821^*^	0.703	1.443	4.199
Learned skills	λ_α_	0.471^*^	0.217	0.046	0.896
Gender	λ_*g*_	−0.350	0.346	−1.028	0.328
Country	λ_*c*_	−0.225	0.296	−0.805	0.355
IV	λ_*IV*_	0.165	0.355	−0.531	0.861
Strategy 1	λ_*st*1_	−0.101	0.255	−0.601	0.399
Strategy 2	λ_*st*2_	0.101	0.443	−0.767	0.969
Gender*IV	λ_*gI*_	0.226	0.312	−0.386	0.838
Country*IV	λ_*cI*_	−0.004	0.285	−0.563	0.555
IV*Strategy 1	λ_*Ist*1_	0.216	0.233	−0.241	0.673
IV*Strategy 2	λ_*Ist*2_	0.280	0.468	−0.637	1.197

IV, Intervention; Dummy coding of the categorical variables: gender (female 1, male −1), Country (US 1, China −1), IV: colored (1), non-colored(-1), Strategy 1: (Compare Analytic Strategy and Holistic Strategy with Hybrid Strategy), Strategy 2: (Compare Analytic with Holistic Strategy);

* *p < 0.05*.

Next, we evaluate the learning program by investigating the overall growth of spatial skills. The output from the dynamic response model indicates that at the initial time point, that is when the participants finished the first testing module and before they received the first learning module, 59.5% participants were estimated as mastery of four rotation skills. Because those participants had already mastered the four skills before receiving the learning modules, we excluded them from the following analysis to better evaluate the learning program. We refer the rest 222 participants who at least missed one rotation skill in the beginning as the non-masters. The overall effectiveness of the learning program is evaluated on summarizing the growth of the non-masters.

##### 5.4.1.1. The overall growth of non-masters

**Figure 11:**
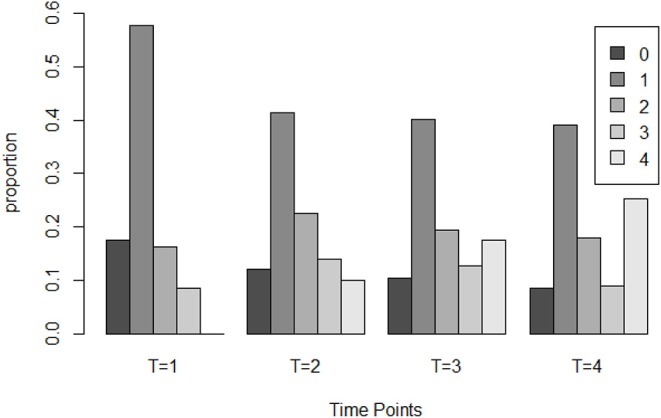
The distribution of the number of mastered skills for non-master group at four time point.

We first report a paired *t* test result that compares the test score from module 1 and module 4, as the items in these two modules have similar psychometric properties and can be treated as a pretest and a post-test. On average, for the non-masters, the module 4 test score (*M* = 6.032, *SD* = 1.780) is significantly higher than the module 1 test score (*M* = 5.716, *SD* = 1.638) and with a small to median effect size, *t*(221) = 2.060, *p* = 0.04, *r* = 0.137. Then the results from the dynamic response model using the item score in the four modules are explored. The overall learning trajectory, denoted as the distribution of the number of mastered skills at each time point, is documented in Figure 11. From there we can observe a “growth” of the rotation skill as the number of non-masters who mastered none of the skills reduced from 17.6% in the beginning of the experiment to 8.5% at the end of the experiment. There are also about 25.2% non-masters mastered four skills in the end. Table 8 further documents the proportion of people who mastered each skill after test module 1 and 4. The results from a χ^2^ test that compares the paired proportion indicates a significant increase of mastery for each skill with medium effect size (Cohen's h). This demonstrates the newly developed learning program can significantly improve the non-masters' four spatial rotation skills.

**Table 8:** The Skill Mastery Rate (proportion of participants that master each skill).

**Skill**	**Time 1**	**Time 4**	**Difference**	***p*-value**	**Cohen's *h***
*x*90	0.812	0.876	0.064	< 0.01	0.290
*x*180	0.648	0.761	0.113	< 0.01	0.374
*y*90	0.761	0.836	0.075	< 0.01	0.301
*y*180	0.628	0.730	0.102	< 0.01	0.322

*Module 1 and Module 4 represent time 1 and time 2*.

**Figure 12:**
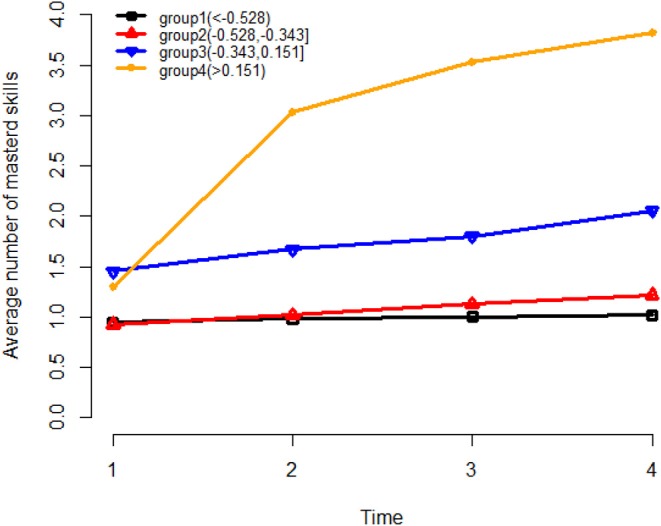
The average number of mastered skills at each time point for four learning ability groups. −0.528, −0.343, and 0.151 are the 1st, 2nd, and 3rd quartile of θ values.

Next, we further investigate how the learning trajectory is influenced by the general learning ability θ. Based on the results from the transition model (Table 7), we can conclude that for a specific rotation skill, the odds of transition from non-mastery to mastery is significantly positively related to the general learning ability θ, $\left({\hat{\lambda }}_{\theta }=2.821,p<0.05\right)$ and the number of mastered skills $\left({\hat{\lambda }}_{\alpha }=0.471,p<0.05\right)$. To further explore these two variables, the non-masters were divided into four groups based on their estimated general learning ability θ. For each group, the number of mastered skills at each stage of the experiment was investigated. The three cut off points were selected as the 1st (−0.528), 2nd (−0.343), and 3rd quantile (0.151) of the estimated general learning ability so that group 1 consists of participants with the lowest learning ability and group 4 consists of participants with the highest learning ability. Figure 12 presents the average number of mastered skills at each time point for each of the four groups. From there we can see that, for the participants with low learning ability (group 1), their learning rate was the lowest. While for the high learning ability participants (group 4), the learning rate is the highest (starts with around 1.5 skills and can master more 3–4 skills). This figure also illustrates how the learned skills can help learn the un-mastered skills. For the participants starting with more than one skills (group 3 and group 4), they learned much faster than the participants starting with 1 or <1 skill (group 1 and group 2).

#### 5.4.2. Dynamic Response Time Model Result

The estimated coefficients for covariates (ϕ) in the dynamic response time model are presented in Table 9. First, on average, the participants who mastered the required skills for an item spent 1.38 s more on completing this question, compared with those who did not master all the required skills (${\hat{\phi }}_{\alpha }=0.327,p<0.05$). Given the participants who had the same learning trajectory, the male participants completed a question faster than female participants (${\hat{\phi }}_{g}=-0.081,p<0.05$); the participants in US completed a question faster than participants from China (${\hat{\phi }}_{c}=0.084,p<0.05$); the participants using colored intervention completed a question faster than participants using non-colored intervention (${\hat{\phi }}_{IV}=0.072,p<0.05$); and finally, the average response time of participants who used analytic strategy and who used holistic strategy were shorter than the one who used a combined strategy (${\hat{\phi }}_{st1}=0.034,p<0.05$), and the participants using a holistic strategy completed a question faster than participants using an analytic strategy (${\hat{\phi }}_{st2}=-0.093,p<0.05$).

**Table 9:** The estimated ϕs from the response time model.

				**95% credible interval**	
**Variable**	**Notation**	**Mean**	***SD***	**Lower bound**	**Upper bound**
*G*(α_*i*_(*t*), q_*j*_)	ϕ_α_	−0.327^*^	0.028	−0.382	−0.272
Gender	ϕ_*g*_	−0.081^*^	0.017	−0.114	−0.048
Country	ϕ_*c*_	0.084^*^	0.016	0.053	0.115
IV	ϕ_*IV*_	0.072^*^	0.023	0.027	0.117
Strategy1	ϕ_*st*1_	0.034^*^	0.012	0.010	0.058
Strategy2	ϕ_*st*2_	−0.093^*^	0.033	−0.158	−0.028
Gender*IV	ϕ_*gI*_	0.017	0.018	−0.018	0.052
Country*IV	ϕ_*cI*_	0.017	0.016	−0.014	0.048
IV*Strategy1	ϕ_*Ist*1_	0.011	0.013	−0.014	0.036
IV*Strategy2	ϕ_*Ist*2_	0.038	0.032	−0.025	0.101

IV, Intervention; Dummy coding of the categorical variables: gender (female 1, male −1), Country (US 1, China −1), IV: colored (1), non-colored(-1), Strategy 1: (Compare Analytic Strategy and Holistic Strategy with Hybrid Strategy), Strategy 2: (Compare Analytic with Holistic Strategy);

* *p < 0.05*.

#### 5.4.3. Survey Questions for Validation

According to the survey collected at the end of experiment, 68% participants rated greater or equal to 3 regarding the questions, “Do you think the learning program is helpful or not.” This question used the 5 points Likert scale with 1 indicates “not very helpful” and 5 denotes “very helpful.”

## 6. Discussion

This study investigated the possibility of developing a learning program that integrates a multidimensional diagnostic assessment with two different learning interventions with the purpose to diagnose and improve the 3-D mental rotation skills. The program was evaluated through an experiment paired with the statistical analysis from a joint model of response accuracy and response times. Compared with the traditional assessment on spatial skills, where the tests are timed and number correct is reported as a measure for test-takers' performances, the proposed diagnostic assessment through the analysis from the joint model can provide an informative estimate of the learning trajectory for each participant in terms of the strengths and weaknesses in four fine-grained mental rotation skills over time. The response times are also utilized to discover additional information about learning across different covariates. While the earlier study (Wang et al., 2017) provided initial evidence of the effectiveness of building a multidimensional diagnostic assessment with training tools, the present study improved the assessment and learning intervention design and evaluated the newly developed program by investigating the effectiveness of two interventions across gender, country and rotation strategy.

The results from the joint learning model demonstrated that learning of a specific rotation skill is significantly related to a general learning ability and the mastered skills. Figure 7 illustrates that it is difficult for test-takers who mastered none of four rotation skills to improve over a short time training. Table 8 indicates the learning of the four rotation skills may follow a hierarchical structure, as the *x*90° rotation might be the easiest one to learn and *y*180° is the most difficult to learn. Thus, to train the test-takers with extremely low spatial ability, it's better to start with a relatively simple and single rotation then transfer to more complex task. This in fact supports the current learning program that first provides an easy learning module then a more challenging one.

However, the current learning program is not adaptive, meaning all the participants received the same learning modules. The results from this study can guide a future design of the adaptive intervention that targets at the weakness of the specific spatial skill and provide the appropriate learning materials. In addition, the output from the dynamic response model portion of the joint model indicates the learning programs with the two designed interventions had the same effectiveness to improve the response accuracy across gender, country and rotation strategy. However, the dynamic response time model reveals the speed difference between the female and male participants, participants using colored and non-colored intervention and participants using three different rotation strategies. Such additional information from the dynamic response time are also helpful in designing an adaptive learning system in the future.

The output of the item parameter estimations from the joint learning model provides new insights into the revised PSVT: R test questions as well. As reviewed in the beginning of this paper, the PSVT:R and revised PSVT: R test questions have been used in many research studies and in generally were reported to have high reliability. The item parameters estimation from the joint learning model indicates that some test questions, especially the ones measure a simple rotation skill can have large guessing parameter, and the ones with complex object and combination of multiple difficult rotation skills may not have good diagnostic information to differentiate the participates with low spatial ability from those with high spatial ability. Carefully examining the distractors may improve their diagnostic functioning. Lastly, another important component in the joint model, is the Q matrix which links the items and the measured attributes. The correct inference from the joint model and the diagnostic assessment relies on how accurate the Q matrix is. The current study used the Q matrix from a previous study, which was mainly specified based on subject experts' opinions. An exploratory clustering method was used to validate the Q matrix, using both response times and response accuracy. It was found that attributes defined in the Q matrix did not contain the information about the degree of complexity of the objects. In the future, we will further validate this Q matrix using many recent techniques in psychometrics (e.g., Chen et al., 2018a).

## Data Availability Statement

The real data set analyzed in this article is not publicly available, because it is part of an ongoing research project from the research team. Requests to access the dataset should be directed to SW, swang44@uga.edu.

## Ethics Statement

The studies involving human participants were reviewed and approved by Institutional Review Board, Office of the Vice President for Research at University of Georgia (IRB ID: STUDY00004215). The patients/participants provided their written informed consent to participate in this study.

## Author Contributions

SW contributed to design the learning program, conducted the experiment, data analysis and draft, and revised the manuscript. YH and BW contributed to design the learning program, conducted the experiment, and drafted the part of the manuscript. QW contributed to help with the design of learning program and conducted the experiment, and also helped to draft the introduction section. YS contributed to the part of data analysis and drafted the part of the manuscript. MC contributed to provide suggestions on designing the learning program.

### Conflict of Interest

The authors declare that the research was conducted in the absence of any commercial or financial relationships that could be construed as a potential conflict of interest.

## Appendix

**Table 1A:** The Wald test results for selecting response measurement model.

**Item**	**Model**	***p*-value**	**Adjusted *p*-value**
Item 1	GDINA	NA	NA
Item 2	GDINA	NA	NA
Item 3	GDINA	NA	NA
Item 4	DINA	0.3407	1
Item 5	GDINA	NA	NA
Item 6	DINO	0.1682	1
Item 7	DINO	0.3288	1
Item 8	RRUM	0.5466	1
Item 9	RRUM	0.3861	1
Item 10	DINA	0.3447	1
Item 11	GDINA	NA	NA
Item 12	GDINA	NA	NA
Item 13	GDINA	NA	NA
Item 14	GDINA	NA	NA
Item 15	GDINA	NA	NA
Item 16	GDINA	NA	NA
Item 17	GDINA	NA	NA
Item 18	GDINA	NA	NA
Item 19	GDINA	NA	NA
Item 20	DINA	0.3018	1
Item 21	GDINA	NA	NA
Item 22	GDINA	NA	NA
Item 23	DINA	0.2031	1
Item 24	GDINA	NA	NA
Item 25	GDINA	NA	NA
Item 26	GDINA	NA	NA
Item 27	ACDM	0.9331	1
Item 28	DINA	0.6384	1
Item 29	RRUM	0.088	0.792
Item 30	RRUM	0.0579	0.6374
Item 31	GDINA	NA	NA
Item 32	GDINA	NA	NA
Item 33	GDINA	NA	NA
Item 34	GDINA	NA	NA
Item 35	DINO	0.7287	1
Item 36	DINA	0.8787	1
Item 37	DINA	0.0974	1
Item 38	DINA	0.7211	1
Item 39	DINA	0.0881	1
Item 40	DINA	0.1309	1

*The p-value and adjusted p-value are from the Wald test between the selected reduced DCM model and GDINA model. Thus, those values are NA for the items are best fitted with GDINA model*.
